# Use of Awake Flexible Fiberoptic Bronchoscopic Nasal Intubation in Secure Airway Management for Reconstructive Surgery in a Pediatric Patient with Burn Contracture of the Neck

**DOI:** 10.1155/2018/8981561

**Published:** 2018-10-21

**Authors:** Tolga Totoz, Kerem Erkalp, Sirin Taskin, Ummahan Dalkilinc, Aysin Selcan

**Affiliations:** ^1^Department of Anesthesiology and Reanimation, Nisantasi University, Istanbul Safak Hospital, Turkey; ^2^Department of Anesthesiology and Reanimation, Health Sciences University, Istanbul Bagcılar Training and Educational Hospital, Turkey; ^3^Department of Anesthesiology and Reanimation, Health Sciences University, Istanbul Haydarpasa Sample Training and Research Hospital, Turkey

## Abstract

Although the use of awake flexible fiberoptic bronchoscopic (FFB) intubation is a well-recognized airway management technique in patients with difficult airway, its use in smaller children with burn contractures or in an uncooperative older child may be challenging. Herein, we report successful management of difficult airway in a 7-year-old boy with burn contracture of the neck, by application of FFB nasal intubation in a stepwise approach, first during an initial preoperative trial phase to increase patient cooperation and then during anesthesia induction for the reconstructive surgery planned for burn scars and contractures. Our findings emphasize the importance of a preplanned algorithm for airway control in secure airway management and feasibility of awake FFB intubation in a pediatric patient with burn contracture of the neck during anesthesia induction for reconstructive surgery. Application of FFB intubation based on a stepwise approach including a trial phase prior to operation day seemed to increase the chance of a successful intubation in our patient in terms of technical expertise and increased patient cooperation and tolerance by enabling familiarity with the procedure.

## 1. Introduction

Secure airway management is crucial in reconstructive surgeries involving patients with burn contracture of the neck [1, 2], while it is a challenge to the anesthesiologist due to anticipation of difficult intubation with likelihood of profound anatomical variation that may not readily be appreciated even during preoperative assessment [1–3].

Awake intubation is considered a safe approach in the airway management of a patient with burn contracture of the neck, particularly for cases presenting with the combination of difficult laryngoscopy as well as difficult mask ventilation [2, 4, 5]. Hence, awake endotracheal intubation remains at the top of the decision algorithm in the recently updated practice guidelines for management of the difficult airway by American Society of Anesthesiology (ASA), although no specific technique or tool has been suggested for accomplishing this task [6].

Fiberoptic-guided tracheal intubation remains the gold standard for pediatric difficult airways and is an essential skill for anyone practicing pediatric anesthesia [7–10]. Awake flexible fiberoptic bronchoscopic (FFB) intubation is a well-recognized airway management technique in patients with difficult airway [4], while its use in smaller children with burn contractures or in an uncooperative older child may be challenging and necessitate inhalational induction technique [2, 11, 12].

Being a cornerstone of safe anesthetic practice in managing patients with identified difficult airway, awake intubation enables prevention of the disastrous consequences of a potential “cannot intubate and cannot oxygenate” scenario, while necessitating a preplanned strategy for intubation and patient preparation regarding explanation of the proposed procedure, sedation, administration of antisialogogues, and regional anesthesia of the airway [2, 4].

Herein, we report successful management of difficult airway in a 7-year-old boy with burn contracture of the neck, by application of awake FFB nasal intubation in a stepwise approach, first during an initial preoperative trial phase to increase patient cooperation and then during anesthesia induction for the reconstructive surgery planned for burn scars and contractures.

## 2. Case Report

**Figure 1:**
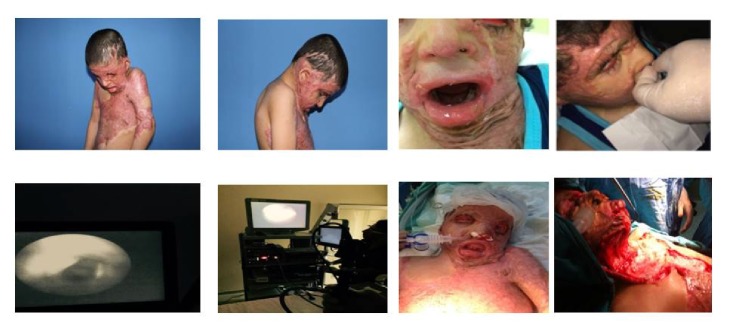
Patient's perioperative images.

A 7-year-old Syrian boy with war-related burn injury was referred to our hospital for reconstructive surgery for burn scars and contractures on his face, neck, and body. A consultation with anesthesia department was held by plastic and reconstructive surgery clinic for the preanesthesia evaluation. Patient was conscious and oriented on examination. He had severe scar contractures involving neck, face, anterior chest, and both shoulders leading to restricted mouth opening, no neck extension, and stooped posture with chin and chest fused together by scars and the neck and head contracted in flexed position. The width from upper incisor to lower teeth was approximately 15 mm and Mallampati class was 3, while thyromental and sternomental distance could not be evaluated due to neck and head being contracted in flexed position. Cardiac, thoracic, and laboratory investigations revealed normal findings. Detailed history of the patient obtained from the parents by the help of a translator revealed that the child had been posted for the reconstructive surgery in another university hospital, while the operation was cancelled due to failure to maintain mask ventilation even after pain relief and induction of anesthesia. The previous anesthesiologist had given two attempts after induction of anesthesia but failed at intubation. Then child was awakened. The day after, he was transferred to our hospital for difficult airway approach and the operation. Awake FFB nasal intubation was planned because of the past history of “cannot intubate and cannot oxygenate” scenario. The necessity and details of the procedure were explained to the patient and his family by the help of a translator. After a 6-hour fasting period, the patient was admitted to our intensive care unit (ICU), accompanied by a family member and translator. Following the routine (NIBP, HR, StO_2_) monitorization (Nihon Kohden, Japan), patient has been informed again about the details and steps of the procedure with the help of the translator. Premedication and sedation were not applied because of the patient's status. During the initial trial phase, nasal drop of xylometazoline 0.1% was instilled for vasoconstriction in both nostrils. Three puffs of 10% lidocaine were implemented for topical anesthesia. Through a nasal cannula, oxygen was administered at 5 L/min through the left side. Tip of the fiberoptic bronchoscope (FOB, 2.8 mm, Karl Storz-Endoskope, Germany) was inserted into the contralateral nostril. Endoscopy was performed. When the vocal cords were visible, the trial procedure was ended. It was explained to the patient and his family that the same procedure would be repeated on the day of surgery as followed by intubation and induction of general anesthesia. On the day of operation, two days after the initial trial, patient was taken to the surgery room and monitored (Infinity Delta Dräger, Lübeck, Germany) routinely (NIBP, HR, SatO_2_). A nasal drop of xylometazoline 0.1% was instilled for vasoconstriction. Three puffs of 10% lidocaine spray were implemented for topical anesthesia. It directly sprayed onto the mucosa of the mouth, pharynx, and tongue. Through a nasal cannula, oxygen was administered at 5 L/min through the left nostril. Endoscopy was performed through the right nostril. Two ml of 2% lignocaine was sprayed through the FOB on to the glottis after the vocal cords were seen. The FOB's tip was then passed into the trachea through the laryngeal opening and was stopped just above the carina. Lubricated 5.0 nasotracheal tube was railroaded over the FOB. After three ventilations, position of nasotracheal tube was confirmed by the FOB. Successful tracheal intubation had been achieved while maintaining spontaneous ventilation and was monitored by capnography. Propofol, fentanyl, and rocuronium were used for induction of general anesthesia via intravenous route and maintained with remifentanil 0.1 *μ*g/kg/min and sevoflurane in oxygen (Primus workstation Dräger, Lübeck, Germany). The operation lasted for approximately four hours. The contractures on neck and left axilla were released and graft was placed. The intraoperative course was uneventful. The patient was extubated after complete recovery of consciousness, adequate spontaneous breathing, preventive reflex, and muscle strength [13] (Figure 1).

## 3. Discussion

Our findings indicate feasibility of awake FFB nasal intubation in a 7-year-old boy with burn contracture of the neck accompanied with restricted mouth opening, no neck extension, and fixed flexion deformity, during anesthesia induction for reconstructive surgery for burn scars and contractures.

Restricted mouth opening, Mallampati class (>2), lack of neck movement, and inability to evaluate thyromental and sternomental distance due to flexed position of neck and head in our patient signify a difficult airway and emphasize that certain airway assessment parameters useful in evaluation of a difficult airway are not applicable in patients with burn contracture of the neck [2, 14].

Given patient's history of difficult tracheal intubation and/or mask ventilation, our findings support the consideration of alternative rather than standard means of securing an airway as a first-line option in patients with face and neck contracture [1, 2]. Our findings also emphasize the utility of awake FFB nasal intubation in postburn pediatric patients with fixed flexion deformity related nonalignment of the oral, pharyngeal, and laryngeal planes for intubation [2, 4], provided that measures to enable sufficient patient cooperation were implied.

Although FFB is considered to be the probably most commonly used tool for awake endotracheal intubation in developed countries [4, 15], in accordance with its consideration as the least traumatic and most efficient alternative to direct laryngoscopy in patients with postburn scar contractures of the neck [1, 16, 17], there are other tools available such as Fastrach intubating laryngeal mask airway (ILMA) [4, 18].

In a past study on comparison of safety and efficacy of ILMA and FFB in awake tracheal intubation of patients with difficult airway, FFB was reported to be associated with significantly lower rate of success on the first attempt (58% versus 95%) along with need for multiple attempts in 42% of patient [4]. However, ILMA has been associated with limitations in certain patients such as those with very restricted mouth opening who need a nasal intubation and those aged <10 years or weighing less than 30 kg due to unavailability of pediatric sizes [4, 19, 20]. Hence FFB seemed to be most reasonable option for management of difficult airway via awake intubation in our patient given his young age and the need for a nasal intubation.

FFB intubation with topical anesthesia of the upper airway in an awake or sedated patient is considered by anesthesiologists as the ultimate, safe, nonsurgical technique in difficult airway management [21]. Although, FFB intubation can cause significant circulatory responses in healthy anesthetized children, the circulatory responses to flexible nasal intubation are considered less frequent and shorter-term responses than those to flexible oral intubation [22].

Hence, based on history of an unsuccessful attempt for intubation and mask ventilation under sedation or general anesthesia in our patient, we preferred awake FFB nasal intubation with use of no sedative or anesthetic agent. While, from a physiologic perspective, children have higher rates of oxygen consumption, significantly shortening the period of apnea that can be safely tolerated [23], no hypoxia occurred during the procedure in our patient given that oxygen was applied through the contralateral nostril.

Although application of fiberoptic tracheal intubation through the nasal route rather than the oral route is considered likely to be more straightforward for clinicians with less experience using pediatric bronchoscopes [6], use of FBB in awake intubation is considered to require significant training and experience to achieve a high success rate [4]. This seems notable given that successful outcome of an awake intubation is considered a net result of different factors including appropriate case selection and good patient preparation to optimize the patient's comfort and compliance as well as technical expertise method of intubation [24].

Awake intubation has been advocated as the safest technique to secure the airway in a cooperative patient for a difficult airway [12, 25]. In this regard, application of FFB nasal intubation based on a stepwise approach including a trial phase prior to operation day seemed to increase the chance of a successful intubation in our patient in terms of improved technical expertise as well as increased patient cooperation and tolerability after familiarity with a procedure limiting the handicaps of a translation-mediated communication.

Our findings support that vigilance and preparedness is the key to success in the postburn patient with crucial role of judicious preoperative airway and scar evaluation to timely recognition of anticipated difficulty with ventilation, intubation, or both in order to develop a preplanned strategy for airway control [1, 2, 14].

Some anesthesiologists have also reserved videolaryngoscopy (VL) for difficult pediatric airway according to the algorithm [26]. On the other hand, FFB intubation is still the gold standard for anticipated difficult airway management in children. Use of VL might have been an alternative [27]. Like our method, awake VL technique also may be considered in children

The limitation of our method is that we did not use short-acting sedatives/opioids, such as remifentanil or midazolam for sedation in this case, because of detrimental effects, such as muscle rigidity, hypoxemia, respiratory arrest, or hemodynamic fluctuations requiring treatment [28], and ketamine or sevoflurane administration during procedure because of awake FFB intubation effort.

In conclusion, our findings emphasize the importance of a preplanned algorithm for airway control in secure airway management and feasibility of awake FFB nasal intubation in a 7-year-old boy with burn contracture of the neck and fixed flexion deformity, during anesthesia induction for reconstructive surgery for burn scars and contractures. Application of FFB intubation via stepwise approach with a trial phase prior to operation day seemed to increase the chance of a successful intubation in our patient in terms of technical expertise and increased patient cooperation and tolerance via familiarity with the procedure. We would also emphasize the importance of preoperative demonstration and explanation of fiberoptic procedure, which is something original and probably resulted in key point for successful maneuver.

## Additional Points

*Highlights*. (i) Use of awake FFB revealed successful airway management in burn contracture of neck. (ii) Preoperative trial FFB seems likely to increase patient cooperation and toleration. (iii) Awake FFB nasal intubation seems feasible in pediatric difficult airway management.
